# FUBP3: A new player in HIV-1 transcriptional activation and immune regulation

**DOI:** 10.1016/j.omtn.2025.102565

**Published:** 2025-05-27

**Authors:** Manukumar Honnayakanahalli Marichannegowda, Mohamed S. Bouzidi, Satish K. Pillai

**Affiliations:** 1 Vitalant Research Institute, 360 Spear St., San Francisco, CA 94105, USA; 2 Department of Laboratory Medicine, University of California, San Francisco, San Francisco, CA, USA

## Main text

**Figure 1:**
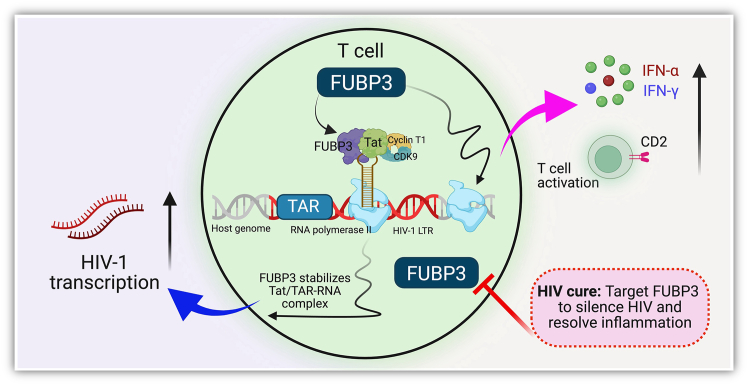
FUBP3 as a dual regulator of HIV-1 transcription and immune response in T cells

Despite effective antiretroviral therapy (ART), HIV-1 infection is not cured due to the persistence of a latent viral reservoir during treatment.^1^^,^^2^ HIV-1 cure strategies therefore focus on eliminating the HIV reservoir to prevent future viral rebound. Alternatively, a recently developed cure framework known as the “block-and-lock” strategy aims to permanently silence latent reservoirs, thereby preventing viral rebound and eventual immunodeficiency.^3^^,^^4^ Identifying host factors that regulate HIV-1 transcription and latency will be critical to the development of effective block-and-lock approaches. A recent detailed investigation published in *Molecular Therapy Nucleic Acids* by Gibaut et al. identifies the far upstream element-binding protein 3 (FUBP3) as a novel enhancer of HIV-1 transcriptional activity and a regulator of immune response pathways in T cells (Figure 1).^5^ This discovery of FUBP3 regulation of HIV-1 transcription may offer a promising new approach to reinforce viral silencing and advance the block-and-lock HIV cure strategy.

The Gibaut et al. investigation uses an innovative chromatin immunoprecipitation combined with mass spectrometry (ChAP-MS) technique, an unbiased approach, to discover FUBP3 as a significant player in HIV transcription. The interaction of FUBP3 with the HIV-1 trans-activator of transcription (Tat) protein was found to enhance viral transcription. Furthermore, the direct binding of FUBP3 to trans-activation response (TAR)-RNA and stabilization based on the presence of Tat were revealed, suggesting a cooperative mechanism for HIV-1 transcription.^6^ Interestingly, the FUBP3-TAR-Tat complex binds to promoters upstream of the transcription start site in the HIV long terminal repeat, which helps RNA polymerase II recruitment and elongation, thereby enhancing transcription.

The authors demonstrate that FUBP3 is a novel host factor that HIV-1 exploits to favor its replication using multiple mechanisms. Transcriptomic analysis showed that FUBP3 depletion could affect the expression of numerous host immune-related genes linked to T cell activation and inflammation. Genes such as CDK1 and KLHL8 and T cell receptor components such as CD2, CCR4, and IL7R were significantly downregulated. Also, the depletion of FUBP3 downregulates the pathways related to tumor necrosis factor alpha signaling via nuclear factor κB, interferon (IFN)-α, and IFN-λ suppression. The finding suggests a dual role of FUBP3 as an enhancer of HIV-1 transcription and an inhibitor of immune response pathways that indirectly suppress HIV transcription.

The Search Tool for the Retrieval of Interacting Genes/Proteins (STRING) protein-protein interaction analysis showed that the genes upregulated by FUBP3 are associated with cancer, cell migration, and nervous system development. Also, quantitative reverse-transcription PCR validated FUBP3-mediated induction of CCR4 and IL7R gene expression, suggesting that FUBP3 regulates the expression of T cell receptors, which influence T cell activation. The analyses of FUBP3 depletion effects on the host transcriptome yielded three key observations: (1) FUBP3 depletion downregulates several genes that are known to be involved in HIV transcription and replication, including CCR1, CCR2, CCR5, CD2, CD28, CD38, CD69, ITGAL, CDK1, and ribonucleotide reductase subunit M2, which plays a vital role in hepatitis B virus and cancer biology by maintaining the deoxyribonucleotide triphosphate pool for DNA biosynthesis and replication^7^^,^^8^; (2) FUBP3 depletion downregulates the transmembrane glycoprotein CD2 at both mRNA and protein levels in all investigated cell lines in the study. CD2 is a commonly found receptor on T cells, natural killer cells, thymocytes, and dendritic cells, which was identified as a cell surface biomarker of HIV latently infected resting CD4^+^ T cells in people living with HIV (PLWHs)^9^; and (3) FUBP3 depletion downregulates the nuclear receptors retinoic-acid-receptor-related orphan receptor β and γ, which are established cellular co-factors that support HIV-1 transcription in lymphoid tissue-resident Th17 cells. Taken together, the study’s findings suggest that FUBP3 directly facilitates HIV transcription and also has a broader impact on a range of host genes responsible for immune response, inflammation, and T cell activation, contributing multifactorially to viral persistence.

Despite promising results, the study also has limitations worth considering: (1) the study was primarily conducted in specific cell lines such as Jurkat and Jurkat-dervied latency (J-Lat) cells, which do not fully represent the complex scenario of HIV infection in primary human CD4^+^ T cells *in vivo* in the presence of multi-lineage cellular interactions; (2) the experiments focus solely on direct and indirect interactions of FUBP3 with HIV transcriptional machinery, but impacts on other aspects of the viral life cycle (e.g., translation of viral proteins or viral budding) are not extensively investigated; and (3) although the study reports that FUBP3 knockdown does not alter HIV-1 integration efficiency, the possibility exists that FUBP3 impacts the distribution of HIV integration sites in the host genome which may exert downstream effects on viral expression and reactivation. Future studies could leverage integration site analysis to reveal whether FUBP3 depletion may shift the integration site toward less transcriptionally active chromatin, which will clarify the observed reduction in HIV-1 gene expression.^10^ Delving deeper into the impacts of FUBP3 on HIV persistence in more physiologically relevant infection models will support a better understanding of the true therapeutic potential of FUBP3 as a pharmacological target, laying a strong foundation for future innovative HIV cure strategies.

In summary, the study by Gibaut et al. demonstrates that inhibiting FUBP3 may represent a novel strategy to suppress HIV-1 transcription, which could provide additional therapeutic value by simultaneously restoring normal immune function in PLWHs. Additional studies are needed to explore the relevance of these findings *in vivo* to determine if targeting of FUBP3 can complement ART and contribute to HIV cure strategies. Importantly, FUBP3 appears to be a broadly active transcriptional regulator and therefore may prove to be a valuable therapeutic target extending beyond HIV into other areas of virology and immunology.

## Acknowledgments

M.H.M. and S.K.P. are supported by National Institutes of Health grants R01AI172754 and R01MH112457, and M.S.B. is supported by the University of California, California HIV/AIDS Research Program (H24BD7806).

## Author contributions

M.H.M., S.K.P., and M.S.B. wrote the manuscript. M.H.M. and S.K.P. created the artwork.

## Declaration of interests

The authors declare no competing interests.
